# Clinical and Hematological Correlation of Nail Changes in Type 2 Diabetes Mellitus: A Retrospective Study

**DOI:** 10.7759/cureus.115801

**Published:** 2026-09-04

**Authors:** Anirudh A Mallapur, Balachandra S Ankad, Manjula Rangappa, Paidela T Reddy

**Affiliations:** 1 Department of Dermatology, S Nijalingappa Medical College and HSK Hospital & Research Centre, Bagalkote, IND; 2 Department of Community Medicine, S Nijalingappa Medical College and HSK Hospital & Research Centre, Bagalkote, IND

**Keywords:** glycated hemoglobin, glycemic control, nail abnormalities, onychomycosis, onychoscopy, type 2 diabetes mellitus

## Abstract

Background

Nail abnormalities are common but often overlooked manifestations of type 2 diabetes mellitus (DM). Their relationship with glycemic control and disease duration remains incompletely understood. This study aimed to evaluate the spectrum of nail abnormalities in patients with type 2 DM and to determine their association with glycemic parameters and duration of DM.

Methods

A retrospective observational study was conducted among patients with type 2 DM attending a tertiary care hospital between January 2023 and December 2024. Patients were divided into two groups: those with nail abnormalities (Group A) and those without nail abnormalities (Group B). Demographic and clinical data, fasting blood sugar (FBS), postprandial blood sugar (PPBS), random blood sugar (RBS), glycated hemoglobin (HbA1c), and duration of DM were analyzed. Nail abnormalities were evaluated clinically and by onychoscopy. Statistical analysis included the Chi-square test, Student’s t-test, correlation analysis, and receiver operating characteristic (ROC) curve analysis.

Results

The study consisted of 100 patients in each group, with Group A (M: 68, F: 32) and Group B (M;52, F: 48). Longitudinal ridging (64%) was the most frequent nail abnormality, followed by onychomycosis (36%), chronic paronychia (26%), Beau’s lines (25%), and nail dystrophy (18%). Patients with nail abnormalities had significantly higher FBS, PPBS, RBS, and HbA1c levels than those without nail abnormalities (all p < 0.001). ROC curve analysis demonstrated that glycemic parameters exhibited superior predictive performance for nail abnormalities compared with the duration of diabetes, with RBS showing the highest sensitivity and HbA1c the highest specificity. Although Beau’s lines were associated with longer disease duration, most nail abnormalities did not demonstrate a significant relationship with the duration of diabetes. Total nail change scores showed a positive correlation with worsening glycemic parameters.

Conclusions

Nail abnormalities in type 2 DM are closely associated with poor glycemic control and may reflect cumulative metabolic injury more closely than the chronological duration of disease. Routine nail examination, supplemented by onychoscopy when indicated, may serve as a simple, non-invasive adjunct for identifying patients who require closer metabolic evaluation. Prospective longitudinal studies are warranted to validate these findings.

## Introduction

Diabetes mellitus (DM) is a common endocrine disorder characterized by chronic metabolic dysregulation and widespread systemic involvement. Its multisystem complications extend to the skin and its appendages, making cutaneous manifestations important indicators of underlying disease burden [1].

Moreover, DM is a heterogeneous disorder comprising multiple subtypes that result from complex interactions between genetic predisposition and environmental factors [2].

Hyperglycemia is associated with a variety of systemic pathological and metabolic alterations that can affect the structure and composition of the nail unit. Nonenzymatic glycation of proteins and lipids results in the formation of advanced glycation end-products (AGEs), which promote oxidative stress and stimulate the release of proinflammatory cytokines. Consequently, DM exerts effects on dermatological health, with nail abnormalities representing important clinical manifestations [3].

Long-standing DM may induce atherosclerotic and microvascular changes in the blood vessels supplying the digits, leading to impaired perfusion and reduced oxygen delivery to the nail matrix. Such vascular compromise can result in alterations in nail morphology, including changes in shape, contour, thickness, and color. Accordingly, nail abnormalities are frequently encountered in patients with diabetes, particularly among those with prolonged disease duration or poor glycemic control. Furthermore, diabetes is characterized by a state of relative immune dysfunction, which predisposes affected individuals to an increased susceptibility to fungal and bacterial nail infections [4,5].

Nail changes may serve as valuable clinical indicators of disease status and chronicity in patients with DM. Despite their potential significance, comparative studies evaluating diabetic patients with and without nail changes remain scarce. Therefore, a study was conducted to evaluate the range of nail changes in patients with DM and to compare them with diabetic patients who do not exhibit nail changes. Additionally, the study aimed to evaluate the relationship between nail changes and disease duration, as well as their association with glycated hemoglobin (HbA1c) levels.

## Materials and methods

This retrospective cohort study was conducted at S. Nijalingappa Medical College and HSK Hospital and Research Center using the hospital diabetes registry maintained in the Medicine Department since 2010. Institutional Ethics Committee approval (SNMC/IECHSR/2021-2022/A-147) was obtained prior to the commencement of the study. Data collected between January 2022 and December 2024 were analyzed. Patients diagnosed with type 2 DM according to the standard criteria were enrolled [6].

All the patients from this registry who are referred to the Dermatology department with complaints of nail abnormalities were included. They are considered as Exposed cohort, i.e., patients with diabetes mellitus and documented nail abnormalities.

Unexposed cohort: Patients with diabetes mellitus without documented nail abnormalities, who are selected from the same hospital registry.

The inclusion criteria consisted of patients with type 2 DM with or without nail changes. Exclusion criteria included patients with type 1 DM, a history of nail trauma, connective tissue disorders, congenital nail abnormalities, and the use of medications known to affect peripheral circulation. Also, patients with inconsistent/incomplete health records and those who did not come for follow-up during the study period were excluded.

Demographic and clinical data, including age, sex, occupation, duration of diabetes, and treatment history, were recorded. Both fingernails and toenails were evaluated in the study. Clinical photographs of affected nails were obtained using a standardized protocol, with a uniform background and adequate illumination. Dermoscopic examination was done using a handheld dermoscope with 10× magnification. Venous blood samples were collected for the estimation of fasting blood sugar (FBS), postprandial blood sugar (PPBS), random blood sugar (RBS), and HbA1c levels.

Sample size: The sample size was calculated based on the expected correlation coefficient (r=0.14) between HbA1c and onychomycosis (OM) reported in a previous study [7].

Assessment of confounding factors: Age and occupation were considered potential confounding variables because nail abnormalities may vary with age and occupational exposure, independently of diabetes-related factors. Age was recorded in completed years, and occupation was categorized using standard classification. However, details of occupational/domestic exposure according to the nature of occupational exposure, particularly activities involving trauma and repetitive use of the hands and feet, were not obtained from the patients. These variables were assessed in both participants with and without diabetic nail abnormalities.

All data were entered into Microsoft Excel and analyzed using Statistical Package for the Social Sciences (SPSS) software version 19.0. Qualitative variables were expressed as frequencies and percentages, while quantitative variables were presented as mean ± standard deviation. The Chi-square test and Student’s t-test were used for the analysis of qualitative and quantitative variables, respectively. Receiver operating characteristic (ROC) curve analysis was performed to evaluate the diagnostic performance of selected parameters. Pearson’s and Spearman’s correlation coefficients were used to assess associations between continuous variables. A p-value of <0.05 was considered statistically significant.

## Results

The study consisted of 100 patients in each group, with Group A (M: 68, F: 32) and Group B (M: 52, F: 48). In Group A, males constituted the majority across all age groups, with the highest proportion observed in the age group between 26 and 50years with 26 (68.4%) patients. Similarly, in Group B, females predominated in the same age group with 38 (59.5%) patients. The demographic characteristics of the study population are summarized in Table 1. Occupational distribution showed a significant difference between group A and group B among unskilled workers, whereas no significant differences were observed for other occupational categories (Table 2).

**Table 1:** Comparison of age distribution between Group A and Group B. Data is represented as numbers and percentages, where percentages are presented in brackets. n: Number

Group	Age (In Years)	Male n (%)	Female n (%)	Total n (%)
Group A	≤25	1 (100.0%)	0 (00.0%)	1 (100.0%)
	26–50	26 (68.4%)	12 (31.6%)	38 (100.0%)
	51-75	38 (66.7%)	19 (33.3%)	57 (100.0%)
	≥76	3 (75.0%)	1 (100.0%)	4 (100.0%)
	Total	68 (68.0%)	32 (25.0%)	100 (100.0%)
Group B	≤25	0 (00.0%)	1 (100.0%)	1 (100.0%)
	26–50	30 (44.1%)	38 (59.5%)	68 (100.0%)
	51-75	21 (70.0%)	9 (30.0%)	30 (100.0%)
	≥76	1 (100.0%)	0 (0.0%)	1 (100.0%)
	Total	52 (52.0%)	48 (38.0%)	100 (100.0%)
Overall	≤25	1 (50.0%)	1 (50.0%)	2 (100.0%)
	26–50	56 (52.8.1%)	50 (47.2%)	106 (100.0%)
	51-75	59 (67.8%)	28 (32.2%)	87 (100.0%)
	≥76	4 (80.0%)	1 (20.0%)	5 (100.0%)
	Total	120 (60.0%)	80 (40.0%)	100 (100.0%)

**Table 2:** Distribution of study subjects according to occupation. Data is represented as numbers and percentages, where percentages are presented in brackets. n: Number

Occupation	Group A n (%)	Group B n (%)
Housewives	31(31.0%)	36 (36.0%)
Professionals	6(6.0%)	6(6.0%)
Unskilled	39(39.0%)	30(30.0%)
Skilled	18(18.0%)	20(20.0%)
Unemployed	6(6.0%)	8(3.0%)
Total	100 (100 %)	100 (100%)

Analysis of nail changes according to the duration of DM demonstrated a significant association between longer disease duration and the presence of specific nail abnormalities. Beau’s lines were more frequently observed in patients with DM duration exceeding 10 years compared with those having disease duration of 1-5 years or 6-10 years. Other nail changes did not show much statistical differences in respect to duration of DM (Table 3).

**Table 3:** Comparison of nail changes by duration of diabetes mellitus. Data is represented as numbers and percentages, where percentages are presented in brackets. The chi-square test is applied as a test of significance, where p value <0.05 is considered as statistically significant. n: Number

Nail changes	Groups	n	Duration of Diabetes						Chi-square	p-value
	Group B		1-5 years		6-10 years		> 10 years			
	Group A		N	%	N	%	N	%		
Beau's Lines	Absent	175	125	96.90%	33	78.60%	17	58.60%	35.594	<0.001
	Present	25	4	3.10%	9	21.40%	12	41.40%		
Chronic paronychia	Absent	174	120	93.00%	33	78.60%	21	72.40%	12.231	0.002
	Present	26	9	7.00%	9	21.40%	8	27.60%		
Onychomycosis	Absent	164	114	88.40%	33	78.60%	17	58.60%	14.622	0.001
	Present	36	15	11.60%	9	21.40%	12	41.40%		
Nail dystrophy	Absent	182	124	96.10%	35	83.30%	23	79.30%	11.988	0.002
	Present	18	5	3.90%	7	16.70%	6	20.70%		
Longitudinal ridging	Absent	136	107	82.90%	16	38.10%	13	44.80%	37.66	<0.001
	Present	64	22	17.10%	26	61.90%	16	55.20%		

Among the nail abnormalities, longitudinal ridging (Figure 1A) was the most common finding and was noted in 64 (64%) patients. OM was noted in 36 (36%) patients. Different types of OM, such as distal lateral subungual onychomycosis (DSLO) (Figure 1C), superficial OM (Figure 2A), total dystrophic OM (Figure 2C) and fungal melanonychia (Figure 3A), were found. Chronic paronychia (Figure 3C), Beau’s lines (Figure 4A), and nail dystrophy (Figure 4C) were observed respectively in 26 (26%), 25 (25%) and 18 (18%) patients.

**Figure 1:**
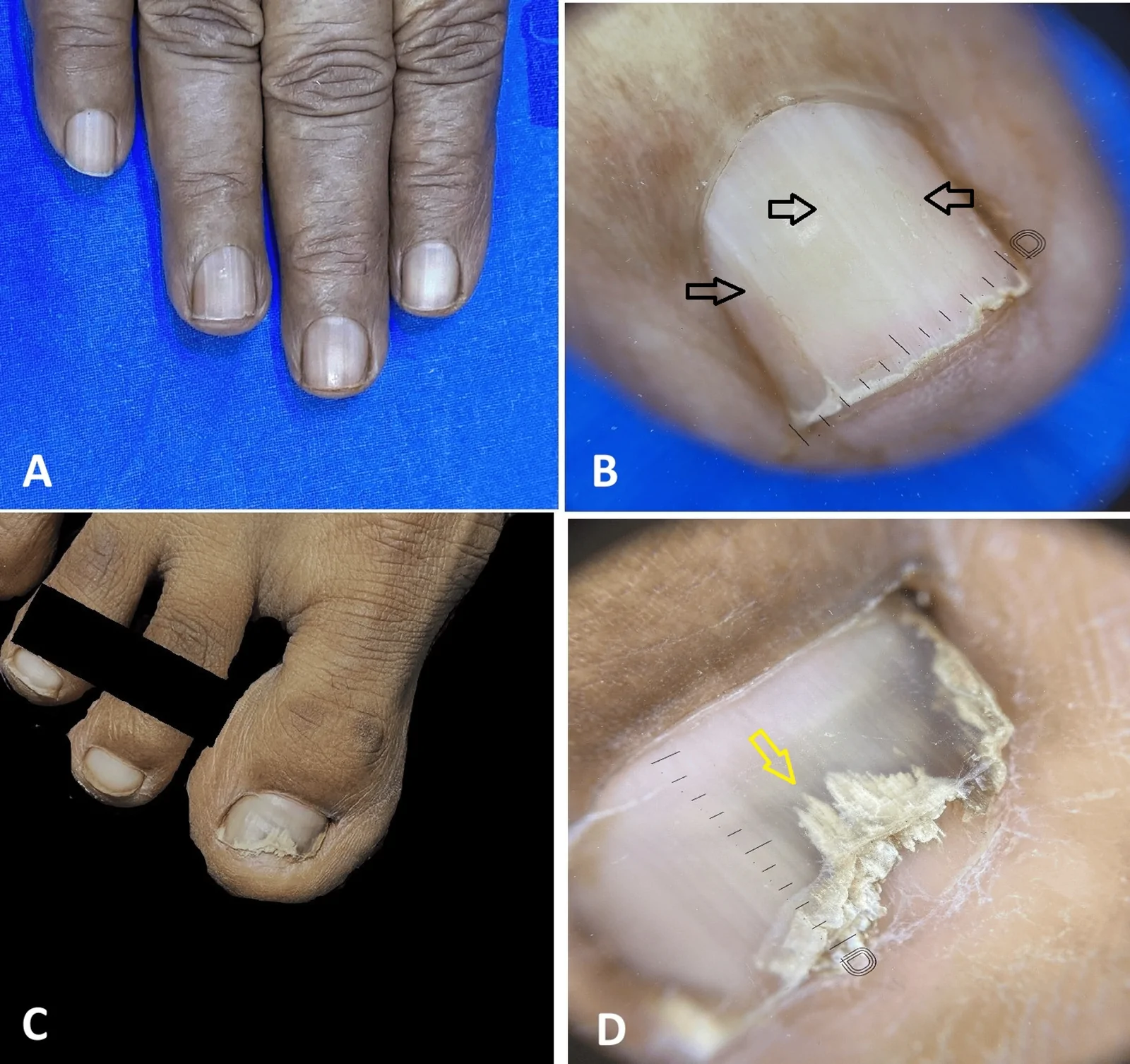
A) Clinical image shows multiple longitudinal ridges on the nail plate. B) Dermoscopy shows linear striations (black arrows). C) Clinical image of distal lateral superficial onychomycosis shows distal onycholysis with breakage of the nail plate. D) Dermoscopy shows yellowish longitudinal striae (yellow arrow) giving the ‘spiked pattern’.

**Figure 2:**
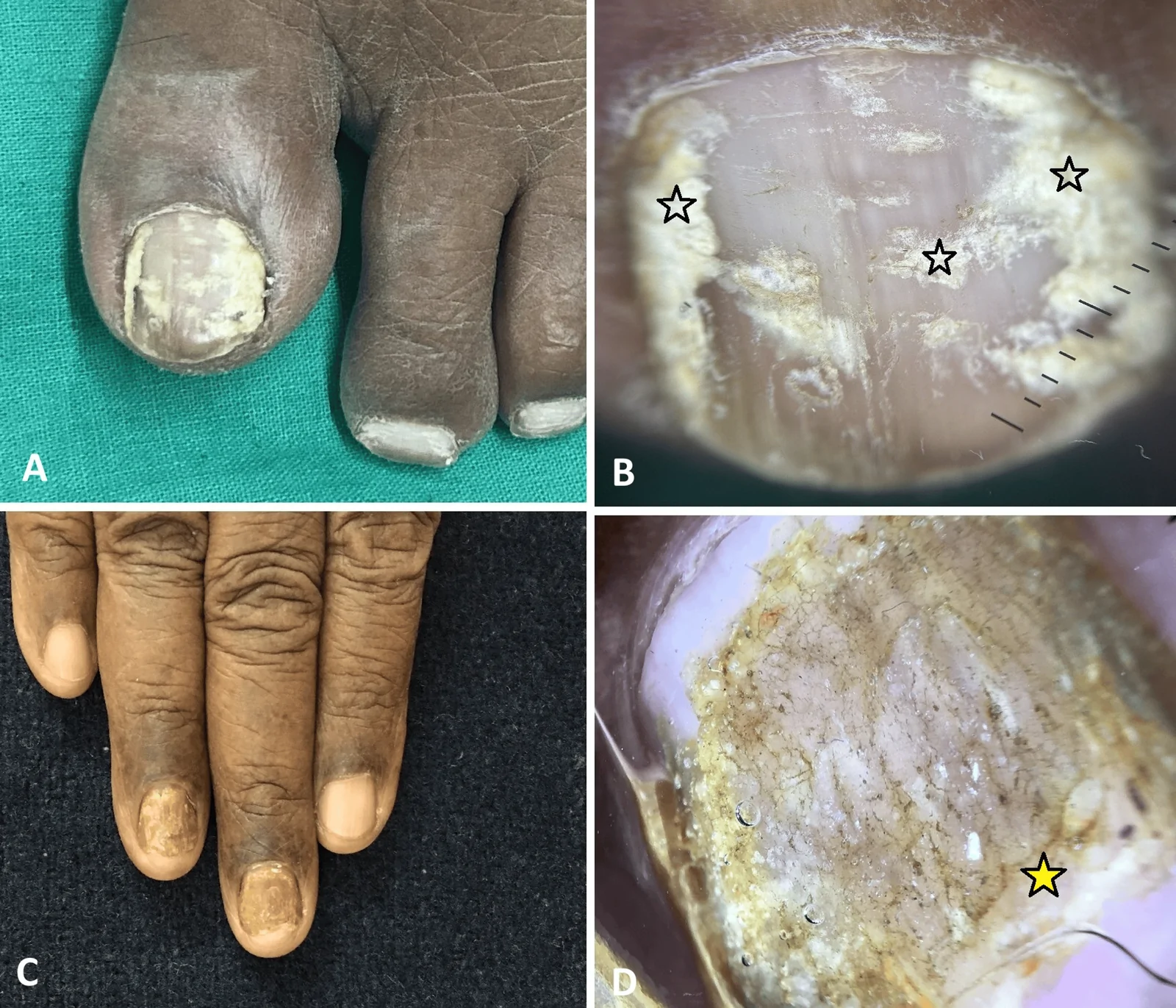
A) Clinical image of superficial onychomycosis shows a whitish-yellow area on the nail plate. B) Dermoscopy shows whitish-yellow areas (black stars). C) Clinical image of total dystrophic onychomycosis shows a roughened, brittle nail plate. D) Dermoscopy shows distal pulverization giving a ‘ruin pattern’ (yellow star).

**Figure 3:**
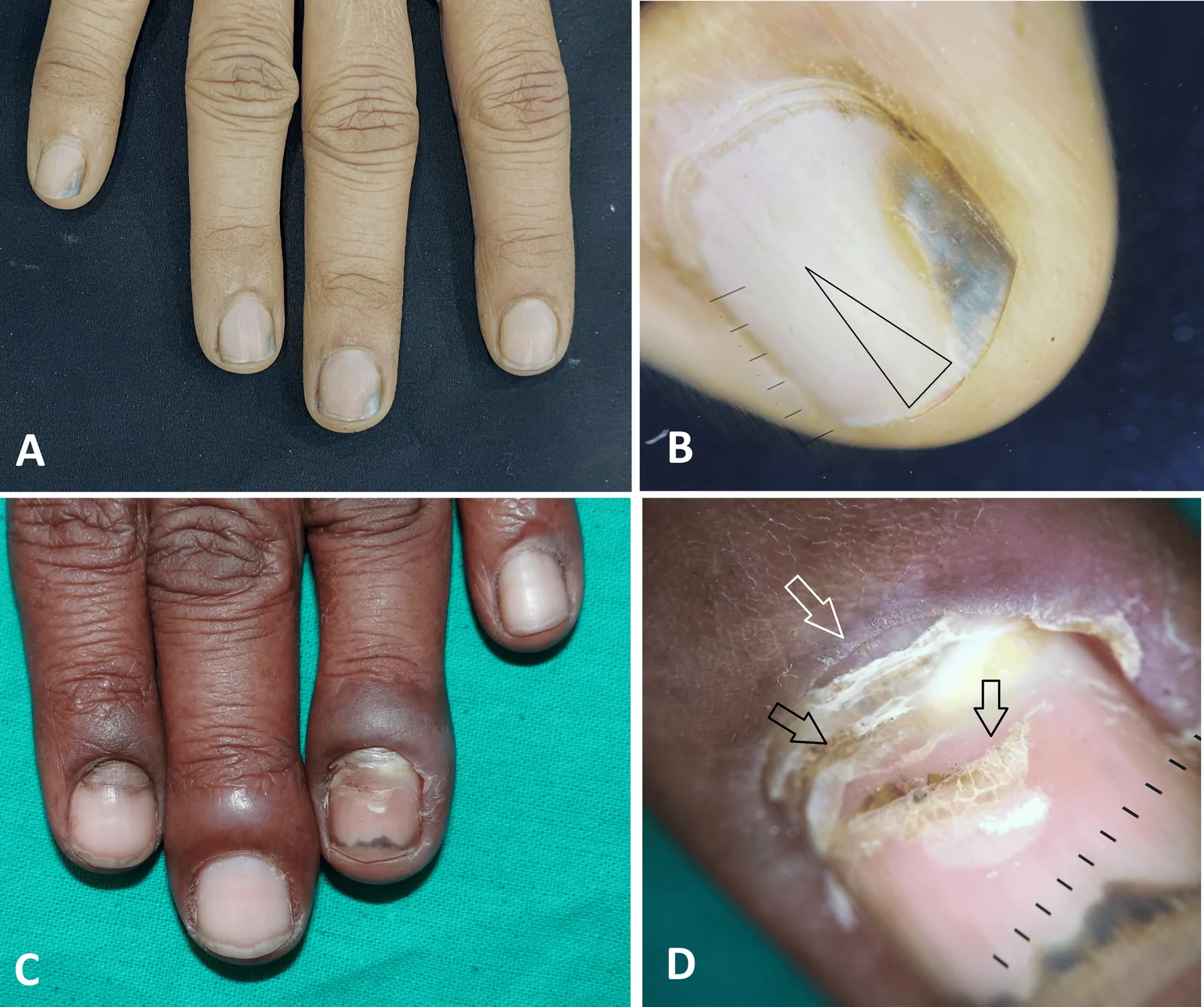
A) Clinical image of fungal melanonychia of onychomycosis shows blackish discoloration of the nail plate. B) Dermoscopy shows a blackish triangular area with a broader base at the distal edge, giving a ‘reverse triangular pattern’ (black triangle). C) Clinical image of chronic paronychia shows swelling in the proximal nail fold and loss of cuticle. D) Dermoscopy shows proximal onycholysis with splitting of the nail plate (black arrows) and loss of cuticle (white arrow).

**Figure 4:**
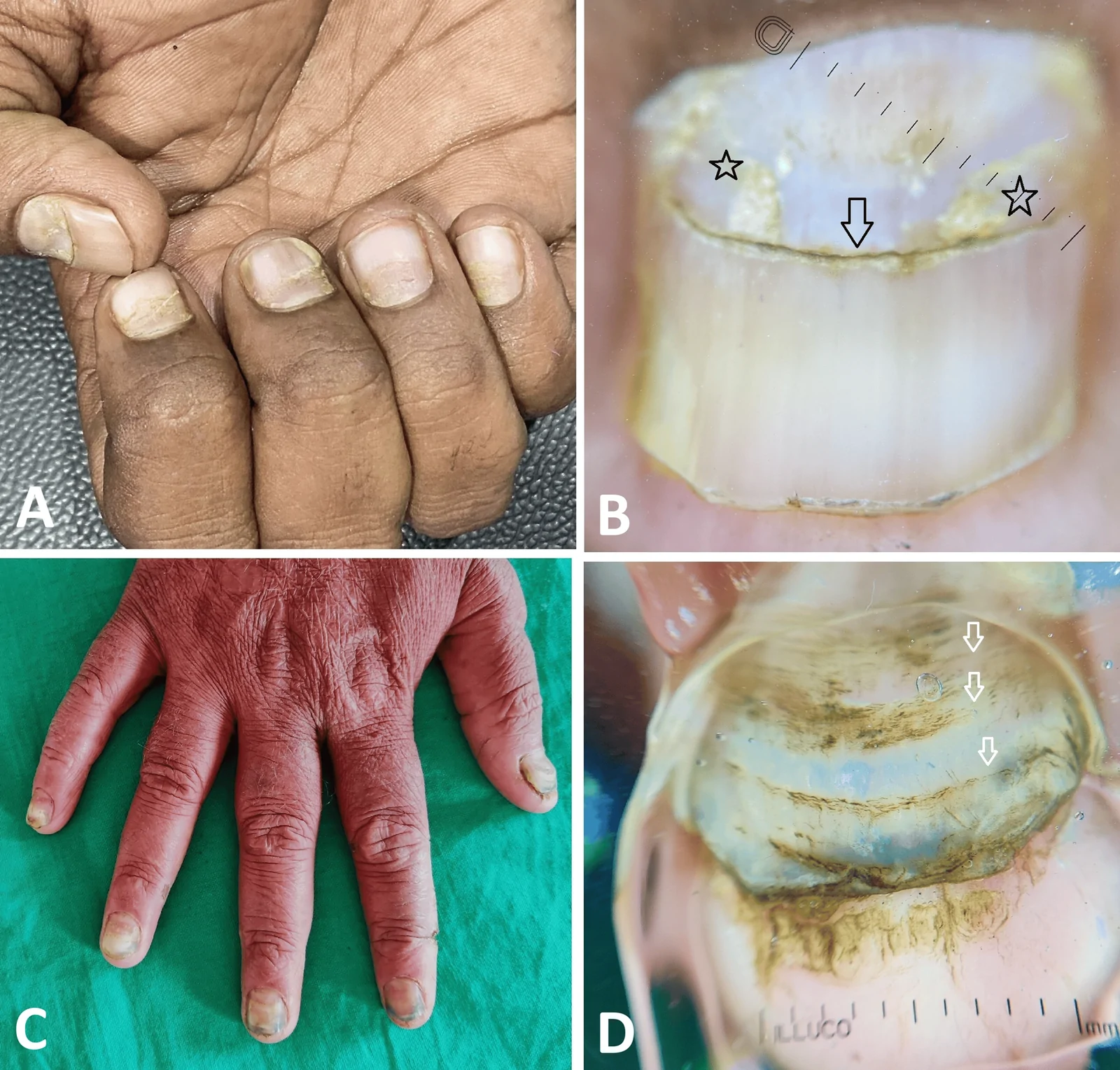
A) Clinical image of Beau’s lines shows regular horizontal ridges on the nail plate in all the fingernails. B) Dermoscopy shows a depressed horizontal line (black arrow) with a few yellow areas (black stars). C) Clinical image of dystrophic nail shows thickened, discolored and brittle nails with surface roughness. D) Dermoscopy shows discoloration, multiple shallow horizontal striations across the nail plate (white arrows).

Onychoscopy demonstrated definitive changes in each entity. Longitudinal ridges appeared as elevated linear striations on the nail plate (Figure 1B). DSLO revealed yellowish longitudinal striae giving the spiked pattern (Figure 1D), and superficial OM showed whitish areas on the nail which represent fungal colonies (Figure 2B). Total dystrophic OM was typified by a ruin pattern wherein distal pulverization of the nail plate was noted (Figure 2D). Fungal melanonychia was represented by a ‘reverse triangle pattern’ in which broader pigmentation is in the distal portion of the nail plate (Figure 3B). Chronic paronychia was characterized by loss of cuticle and disintegration of the proximal nail plate (Figure 3D), and Beau’s lines showed horizontal depressed lines across the nail plate with a few yellow areas, which indicate surface keratin of the nail plate (Figure 4B). Finally, nail dystrophy was revealed as a thickened nail plate, discoloration and multiple horizontal superficial ridges (Figure 4D).

Comparison of clinical and baseline laboratory parameters between Group A and Group B revealed statistically significant differences in FBS, PPBS, RBS, and HbA1c levels (p < 0.001). However, no significant difference in age was observed between the groups (p = 0.068) (Table 4).

**Table 4:** Correlation of baseline hematological parameters with nail changes. Data are represented as Mean and Standard deviation. Student's t-test was applied as the test of significance, where p-value < 0.05 was considered statistically significant. FBS: Fasting blood sugar; PPBS: Postprandial blood sugar; RBS: Random blood sugar; HbA1c: Glycated hemoglobin; Std. Deviation: Standard deviation; T- Student test

Parameters	Group	Mean	Std. Deviation	T	p
	Group B				
	Group A				
Age	No Nail Changes	50.02	14.663	-1.832	0.068
	With Nail Changes	53.55	12.502		
FBS	No Nail Changes	99.701	27.5111	-12.046	0.001
	With Nail Changes	198.016	76.4063		
PPBS	No Nail Changes	118.414	40.7907	-14.546	0.001
	With Nail Changes	275.372	99.3966		
RBS	No Nail Changes	111.465	53.7751	-15.463	0.001
	With Nail Changes	295.218	105.4353		
HbA1C	No Nail Changes	4.912	1.0328	-16.114	0.001
	With Nail Changes	8.715	2.1219		

ROC curve analysis demonstrated that nail changes were significantly associated with poor glycemic control (Figure 5). Duration and hematological parameters had an impact on nail changes. RBS and HbA1c were more sensitive and specific, respectively, to nail changes (Table 5). Correlation analysis between individual nail changes and total nail change scores is presented in Table 6. HbA1c (0.753) had the strongest correlation, and duration (0.483) had a moderate correlation in nail changes in DM. Assessment of confounding factors such as age and occupation was found to be not statistically significant.

**Figure 5:**
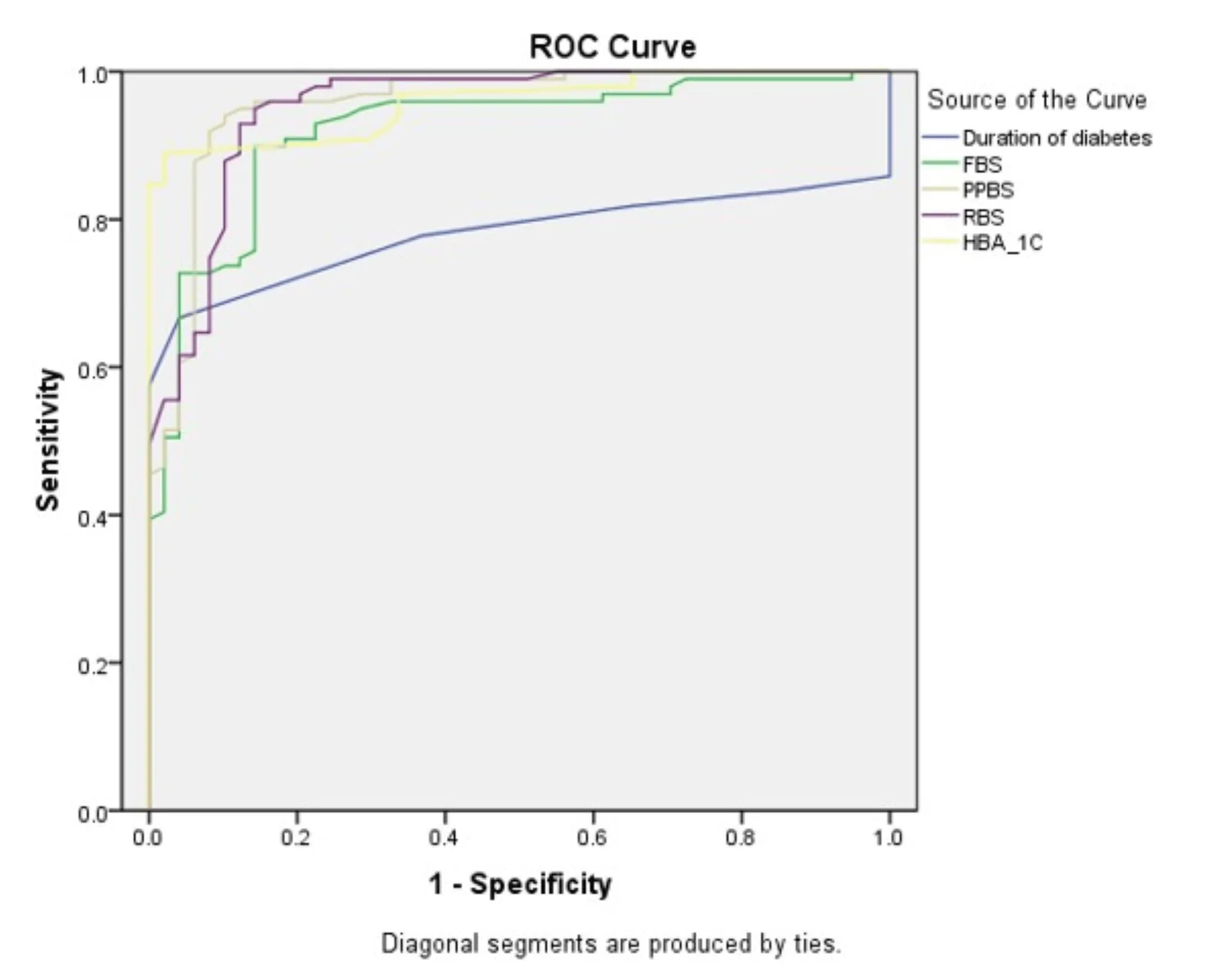
ROC analysis of glycemic parameters and duration of diabetes in predicting nail changes. ROC: Receiver operating characteristics; FBS: Fasting blood sugar; PPBS: Post-prandial blood sugar; RBS: Random blood sugar; HbA1c: Glycated hemoglobin

**Table 5:** Sensitivity and specificity of nail changes in relation to duration of diabetes mellitus and hematological parameters FBS: Fasting blood sugar; PPBS: Postprandial blood sugar; RBS: Random blood sugar; HbA1c: Glycated hemoglobin

Test Result Variable(s)	Area Under the Curve	Criterion value	Sensitivity	Specificity
Duration of diabetes	0.779	>5 years	67%	96%
FBS	0.92	>112mg/dl,	89.90%	85.90%
PPBS	0.956	>143mg/dl,	92%	91.90%
RBS	0.951	>136.9mg/dl,	93%	87.90%
HbA1c	0.958	>6	89%	98%

**Table 6:** Correlation between and nail changes and total nail changes score with different parameters. Pearson’s Correlation test is applied, where r= Correlation co-efficient.  where p-value <0.05 is considered statistically significant. Spearman’s Correlation test was applied, where rho= Correlation co-efficient. where p-value < 0.05 is considered statistically significant. FBS: Fasting blood sugar; PPBS: Post-prandial blood sugar; RBS: Random blood sugar; HbA1c: glycated hemoglobin ** statistically very significant

		Duration of diabetes	FBS	PPBS	RBS	HbA1c
Total Nail changes (score)	r (Pearson’s Correlation coefficient)	0.430^**^	0.590^**^	0.624^**^	0.577^**^	0.598^**^
	P	0.000	0.000	0.000	0.000	0.000
Nail changes	rho (Spearman’s Correlation coefficient)	0.483^**^	0.652^**^	0.720^**^	0.740^**^	0.753^**^
	P	0.000	0.000	0.000	0.000	0.000

## Discussion

The pathogenesis of nail changes in DM is multifactorial. Chronic hyperglycemia leads to the accumulation of AGEs, which trigger oxidative stress and proinflammatory cytokine production. Concurrently, diabetic microangiopathy impairs blood supply and oxygen delivery to the nail matrix, resulting in abnormalities of nail growth and structure. In addition, diabetes-associated immune dysfunction increases susceptibility to infectious nail disorders, particularly fungal infections. Together, these mechanisms contribute to the high prevalence and varied presentation of nail changes in patients with DM [8-10].

Beau’s lines

Beau’s lines are transverse depressions of the nail plate that reflect a temporary arrest of nail matrix activity secondary to systemic stress or illness. In the present study, Beau’s lines were observed in 12.5% of patients and were more frequently associated with longer duration of DM. In contrast, Mahajan et al. reported a prevalence of only 1% [4]. The development of Beau’s lines in patients with DM may be attributed to diabetic microangiopathy and impaired vascular supply to the nail matrix, resulting in transient disruption of nail growth during periods of metabolic stress or poor glycemic control.

Chronic paronychia

Chronic paronychia was observed in 26% of the study population as compared to16% of study subjects in a report [4]. Hyperglycemia, impaired immune function, and disruption of the cuticle barrier facilitate colonization by bacterial and fungal organisms, leading to chronic inflammation of the proximal and lateral nail folds. The absence of an intact cuticle permits the penetration of irritants, allergens, and microorganisms, thereby perpetuating the inflammatory process [11]. Management should focus on optimization of glycemic control, maintenance of hand and foot hygiene, avoidance of prolonged moisture exposure, and restoration of the cuticular barrier. Topical anti-inflammatory agents, including corticosteroids and tacrolimus, may be effective in reducing inflammation and promoting resolution [12]. Interestingly, frequency did not show variation with duration of DM. It raises the possibility that current or cumulative metabolic dysregulation may be more relevant to periungual inflammatory changes than chronological disease duration alone.

Onychomycosis

Onychomycosis (OM) is one of the most frequently encountered nail disorders in patients with DM, with a prevalence significantly higher than that observed in the general population. In our study, the prevalence of onychomycosis was significantly higher among patients with DM (29%) than among healthy controls (11%). A lower prevalence of OM in type 1 DM and a predominance of toenail involvement over fingernail involvement suggest that the development of OM is influenced by the type and duration of diabetes, as well as the metabolic status [13]. Reduced antigen presentation, diminished immune surveillance, and increased susceptibility to infections are attributed to OM [5,14-16].

Distal lateral subungual onychomycosis (DLSO) is the most common clinical subtype of onychomycosis observed in patients with DM [17-19]. Similarly, DLSO was the predominant pattern in our study; furthermore, types of superficial OM, total dystrophic OM and fungal melanonychia were observed as well. However, duration of DM did not have an impact on OM, which could be due to small sample size and variations in the sugar levels among patients in each category of duration of DM.

Nail dystrophy

Clinically, nail dystrophy may manifest as longitudinal ridging, pitting, lamellar splitting, surface peeling, brittleness, and crumbling of the nail plate. Previous studies have reported a prevalence of nail dystrophy ranging from 46% to 54% among patients with DM. The occurrence and severity of nail dystrophy have been shown to correlate with metabolic control, disease duration, age, and sex [8]. In the present study, a similar association was observed with most of these factors; however, no significant relationship with the duration of DM was identified. This discrepancy may be attributable to the relatively small sample size of the study.

Longitudinal ridging

Longitudinal ridging was the most common nail abnormality observed in the present study, occurring in 64% of cases. Similar findings have been reported in previous studies, with Mahajan et al. documenting longitudinal ridging in 46% of diabetic patients. These observations highlight the potential role of longitudinal ridging as a marker of chronic diabetic nail involvement [4,8,10]. The development of longitudinal ridging in patients with DM is thought to be related to chronic hyperglycemia-induced microvascular compromise and the accumulation of AGEs. However, because longitudinal ridging did not show a significant association with duration of DM, it would be inappropriate to interpret this finding as a direct marker of cumulative chronological disease duration. Instead, it may reflect the influence of metabolic exposure and other patient-specific factors that are not captured by disease duration alone.

Relationship between glycemic control and nail abnormalities

An important finding of the present study was the significantly higher FBS, PPBS, and HbA1c levels among diabetic patients with nail abnormalities compared with those without nail abnormalities. The particularly relevant finding of elevated HbA1c is noteworthy because HbA1c reflects average glycemic exposure over a substantially longer period than a single blood glucose measurement. The consistent association of multiple glycemic parameters with the presence of nail abnormalities therefore supports the possibility that poor glycemic control is associated with a greater burden of nail involvement in patients with DM.

ROC curve analysis was performed to assess the ability of the duration of diabetes mellitus and glycemic parameters to discriminate between patients with and without nail abnormalities. Increasing duration of DM and elevated blood glucose parameters beyond their respective optimal cutoff values demonstrated significant discriminatory ability for the presence of nail changes, as evidenced by the area under the curve (AUC) and corresponding 95% confidence intervals. These findings suggest that longer duration of DM and poorer glycemic control are associated with a higher likelihood of nail abnormalities. Overall, the ROC analysis suggested that glycemic parameters demonstrated greater discriminatory ability for the presence of nail changes than the duration of DM.

Furthermore, a positive correlation was observed between the total nail change score and glycemic parameters. This relationship suggests that the severity of nail involvement increases with worsening metabolic control. Therefore, early diagnosis, strict glycemic control, and regular monitoring may help reduce the occurrence and progression of nail changes in patients with DM.

A notable finding of the present study was that glycemic parameters demonstrated better predictive performance for nail abnormalities than the duration of DM. This suggests that metabolic control may have a greater influence on the development of nail changes than the duration of disease. Since disease duration is an indirect measure and may not accurately reflect cumulative glycemic exposure, glycemic parameters could represent a more clinically meaningful indicator of the risk of diabetes-associated nail abnormalities. Nevertheless, this finding should be interpreted cautiously and confirmed in prospective longitudinal studies.

The limitations of the study are its retrospective design and relatively small sample size. In addition, correlations between individual nail changes and glycemic parameters were not evaluated, and the various subtypes of onychomycosis (OM) and paronychia were not analyzed separately with respect to diabetes duration and glycemic control. Lack of microbiological confirmation of the diagnosis of OM and multivariable analysis.

## Conclusions

The present study reflects the possibility of association of nail abnormalities in type 2 DM could be due to the cumulative metabolic burden of the disease rather than its chronological duration alone. Factors such as age and occupation may act as compounding variables, though they were found to be not statistically significant in the study. Routine nail examination, supplemented by onychoscopy when clinically indicated, may therefore serve as a simple, non-invasive adjunct for the early recognition and characterization of diabetes-associated nail abnormalities. These findings further emphasize the importance of optimal glycemic control in potentially reducing the burden of diabetes-related nail disorders and support the incorporation of routine nail assessment into comprehensive diabetic care.
